# Prognostic factors in laser treatment of upper urinary tract urothelial tumours

**Published:** 2012-03-05

**Authors:** Gh Niţă, D Georgescu, R Mulţescu, M Draguţescu, B Mihai, B Geavlete, C Persu, P Geavlete

**Affiliations:** “Sf. Ioan” Clinical Hospital, Bucharest

**Keywords:** laser, upper TCC, urothelial tumour, endoscopic treatment

## Abstract

**Introduction.** The standard treatment for upper urinary tract urothelial cell carcinoma (UUT-UCCs) is radical nephroureterectomy with bladder cuff excision. The endoscopic treatment was introduced with promising results in selected cases. The purpose of this study was the retrospective analysis of the factors that can influence the prognosis of the patients with UUT-UCCs who underwent endoscopic treatment.

**Patients and method.** We identified 187 patients who where diagnosed and treated for UUT-UCCs, between 1998 – 2011, in the Urology Department of “Sf. Ioan” Clinical Emergency Hospital, Bucharest. The endoscopic treatment was used in 65 cases. The indications for endoscopic treatment were imperative (41 cases) or elective (24 cases). The retrograde approach (rigid or flexible) was used in 47 cases, while the anterograde approach was preferred in 18 cases. Tumor ablation was performed using electroresection or Nd:YAG laser. The mean follow-up period was 60 months (range between 6 and 120 months). The follow-up protocol included computed tomography or intravenous urography, urinary cytology (selected cases), cystoscopy and ureteroscopy. The recurrence rates were reviewed by retrospective analysis.

**Results.** During follow-up 31 patients (47.6%) presented upper urinary tract recurrence. In 20 cases (30.7%) bladder recurrence was present. The median time from diagnosis to first recurrence was of 12.6 months. 18 patients (27.69%) underwent subsequent nephroureterectomy. The survival rates without recurrence at 1, 3 and 5 years were 61% (40 patients), 55.3% (36 patients) and 52.3% (34 patients). The most significant prognostic factors were: history of bladder tumour, tumour location and size, tumour stage and grade. The recurrence rate for pyelocaliceal tumours was 53.84% (21 out of 39 cases) and only 45.45% (10 out of 26 cases) for ureteral tumours. The recurrence rate for low-grade tumours was 36,36% (16 out of 44 cases) and 71.42% (15 out of 21 cases) for high-grade tumours. The tumours over 1.5 cm were associated with a higher recurrence rate compared with tumours below 1.5 cm (64.2 versus 43.13%).

**Conclusions.** Endoscopic management of UUT-UCCs offers the advantage of preserving of renal function. Laser treatment of malignant urothelial lesions in the upper urinary tract should be reserved for a selected patient. The most important prognostic factors for UUT-UCCs evolution are tumours location, size and mostly tumour grade. The patients’ compliance is very important for detecting recurrences.

**Abbreviations** UUT-UCCs - Upper urinary tract urothelial cell carcinomas

## Introduction

Upper urinary tract urothelial cell carcinomas (UUT-UCCs) are rare, representing 5 to 10% from all urothelial tumours [**1**]. The annual estimated incidence is 1-2 new cases / 100.000 inhabitants. The pyelocaliceal location is 2 times more frequent than the uretheral one; synchronous bladder tumours are present in 8-13% cases [**2**]. The large majority are transitional carcinomas (90%), while only 10% are squamous carcinomas and 1% are adenocarcinomas. The upper urinary tract transitional carcinomas increase with age in both genders, with a maximum in the sixth and seventh decades. 

**Fig. 1:**
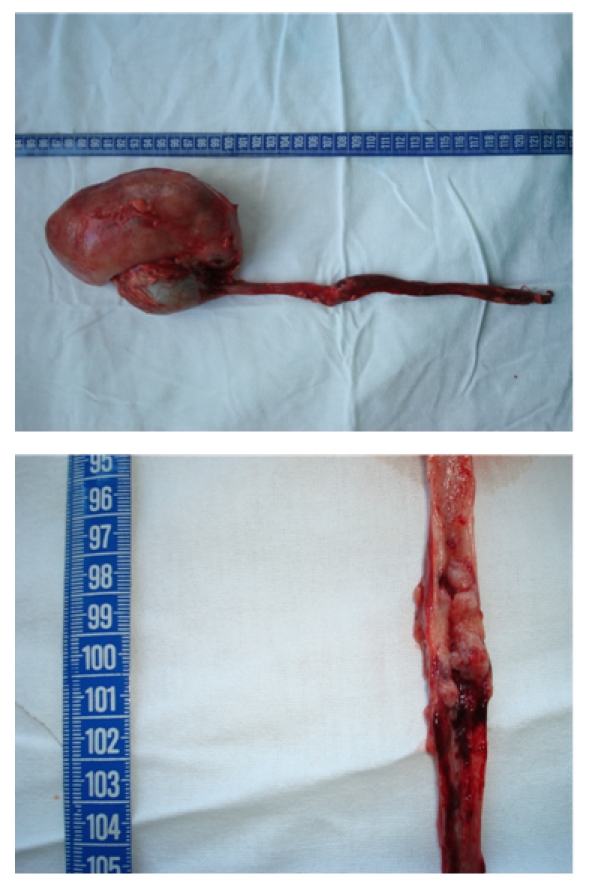
Nephroureterectomy (with endoscopic desinsertion) for ureteral tumours

The standard treatment is radical nephroureterectomy with bladder cuff excision. The operation can be performed in classical fashion (two incisions - lumbar and iliac) or by endoscopic desinsertion of the distal ureter followed by open surgery [**3,4,5**] (**Fig. 1**). The laparoscopic nephroureterectomy, recently introduced, has shown promising results [**6**]. Nevertheless, the risk of tumour dissemination was not totally reduced [**7**].

Despite all this, with the advancements made in fiber optics technology, lasers and smaller caliber endoscopic instruments, the first intention conservative endoscopic treatment was introduced in UUT-UCCs with promising results [**8-14**]. The ureteroscopic and/or percutaneous approach has imperative indications (in patients with solitary kidney, bilateral disease or kidney failure) [**10,15,16**] or elective (superficial single small tumour, in patients with normal controlateral kidney) [**8,9,13**].

The purpose of this study was the retrospective analysis of the factors that can influence the prognosis of the patients with UUT-UCCs who underwent endoscopic treatment.

## Patients and methods

Between 1998 and 2011, a number of 187 patients were diagnosed and treated for UUT-UCCs in the Urology Department of “Sf. Ioan” Clinical Emergency Hospital Bucharest. In 65 cases the endoscopic approach (ureteroscopic or percutaneous) was used as first line of treatment.

**Fig. 2:**
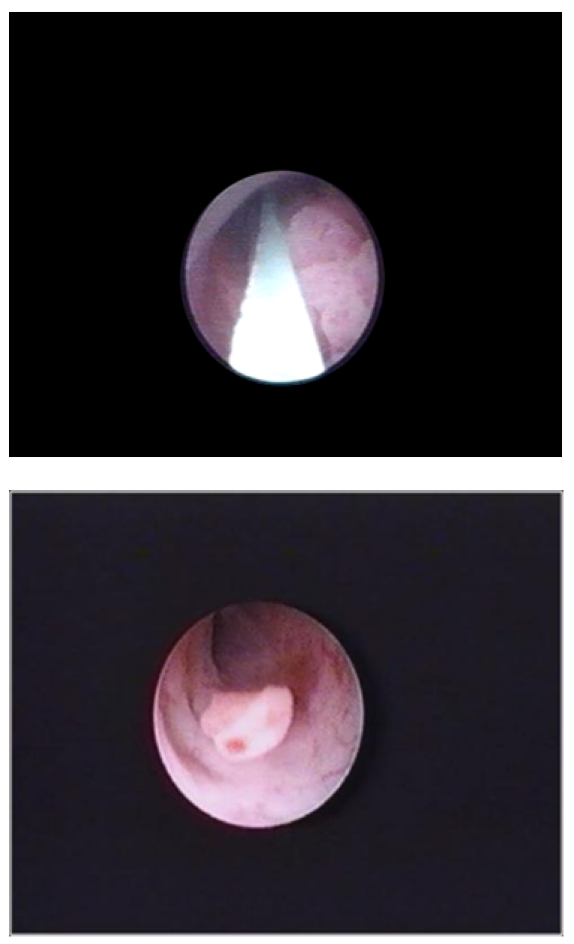
Ureteral tumour with elective indication for endoscopic treatment

The indications for endoscopic treatment were imperative (41 cases) or elective (24 cases). The endoscopic approach was imperative in patients with: solitary kidney (17 cases), bilateral disease (4 cases), and chronic kidney failure with preoperative serum creatinine higher than 2 (9 cases) or other comorbidities (ASA score higher or equal to 3) who were not candidates for open surgery (11 cases). The other 24 cases (with elective indications) had normal contralateral kidney, solitary tumours smaller than 2 cm and visually non-invasive, with no personal history of urinary tract interventions (**Fig. 2**). All the patients in this last group accepted a thorough postoperative follow-up. Patients who could not fulfill these criteria or who refused endoscopic treatment underwent open nephroureterectomy. 

None of the patients included in this study had metastatic disease at the moment of diagnosis. Tumour stage and grading were established according to TNM [**17**] and WHO classifications [**18**]. The preoperative hydronephrosis grade was established by abdominal ultrasound or computed tomography. 

**Fig. 3:**
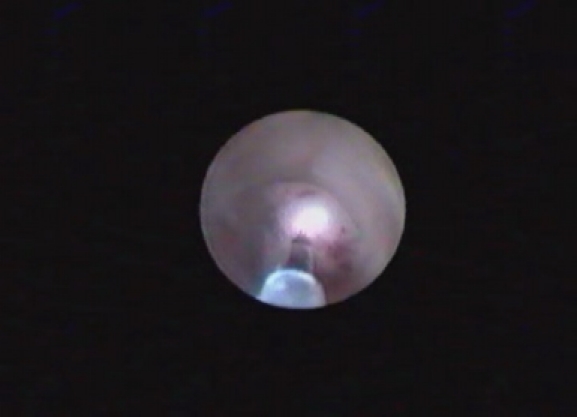
Nd:YAG laser coagulation for ureteral tumour

**Fig. 4:**
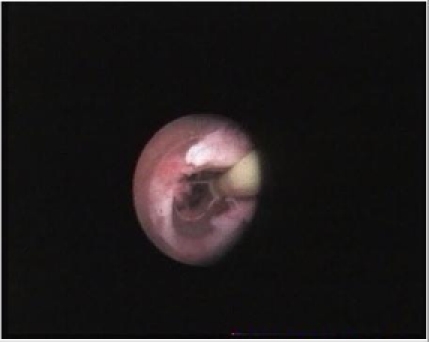
Electroresection for ureteral tumour

When possible, flexible or rigid ureteroscopy (47 cases) was preferred to the percutaneous endoscopic approach (18 cases). The ureteroscopic technique was that described by most authors [**8,19,20**]. Electroresection or coagulation with Neodymium: Yttrium-Aluminium-Garnet (Nd:YAG) laser with 600 or 200μm fibers were used (**Fig. 3and Fig. 4**). For pyelocaliceal tumours higher than 1cm and/or unavailable by ureteroscopy, the anterograde percutaneous approach was used, with the classical technique [**14,19,21**]. The anterograde percutaneous approach included retrograde renal cavities pielography, calix puncture, atraumatic tract dilatation to avoid extravasation of the contrast fluid and the usage of the Amplatz sheath to reduce the intrarenal pressure. Electroresection or Nd:YAG (with power settings between 20 - 45 W) laser were used. The nephrostomy tube was placed for 2-5 days. Two patients received adjuvant topical treatment with mitomicine C, and 6 patients underwent BCG treatment.

The mean follow-up period was of 60 months (range between 6 and 120 months). Patients were evaluated at 3 and 6 months by computed tomography or intravenous urography, urinary cytology (selected cases) and ureteroscopy. In the absence of recurrences, this protocol was repeated at 6 months in the first 2 years and then yearly.

The disease free survival rate and the disease specific survival rate were analyzed. Recurrence after endoscopic treatment was defined as in situ (at tumour site) or local (other upper urinary tract location that underwent treatment). The recurrence was assessed from the date of surgery. The survival without disease was defined as the period of time from the time of surgery till the first local recurrence (urinary tract urothelial tumour), detection of metastasis or end of study. The prognostic factors established by univariate analysis were: age, gender, smoking history, personal history of bladder tumour, grade of hyfronephrosys, tumor location, size, stage and grade.

## Results

The mean age at the moment of diagnosis was of 67 years (range 42-89). Most patients (49 cases, 75.3%) had a smoking history. About 14 % of patients (9 cases) had a personal history of bladder tumour. 

**Fig. 5:**
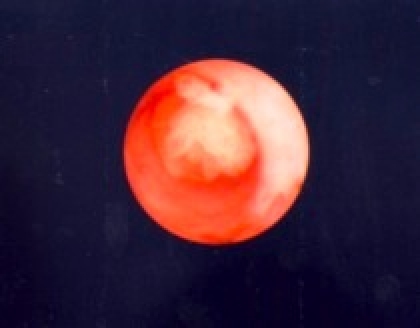
Inferior caliceal tumour

In 39 cases (60%), the tumour was located in the renal pelvis or calices (Fig. 5), while 26 patients had ureteral tumours (40%). For the ureteral tumours, hydronephrosis was absent in 5 cases (19.23%), hydronephrosis was of grade I - II in 18 patients (69.23)%) and grade III in 3 patients (11.54%). All the patients with grade III hydronephrosis had an imperative indication for endoscopic treatment.

47 patients had solitary tumours and 18 patients had multiple tumours. All the patients with multiple tumours had imperative indications for endoscopic surgery.

**Fig. 6:**
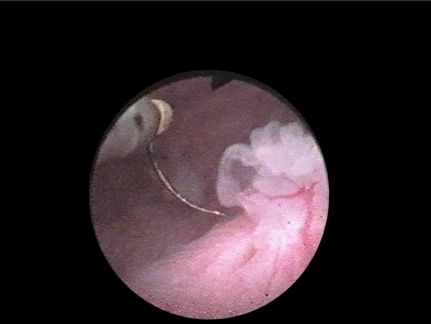
Bladder recurrence after UUT-UCCs

During the follow-up, 31 patients (47.6%) developed upper urinary tract recurrence. In 20 cases (30.7%) the bladder recurrence was noted (Fig. 6). The median time from the moment of diagnosis to the first local recurrence was of 12.6 months. 18 patients (27.69%) underwent subsequent nephroureterectomy.

The disease free survival rates at 1, 3 and 5 years from diagnosis were of 61% (40 patients) , 55.3% (36 patients) and 52.3% (34 patients). 9 patients from those who had recurrence died from TCC. Kidney sparing was accomplished in almost 2/3 of cases, while 18 patients underwent subsequent nephroureterectomy due to disease recurrence. 

In this study, the tumour location, grade, stage, size and the grade of hydronephrosis were the most significant prognostic factors. The recurrence rate for pyelocaliceal tumours was of 53.84% (21 of 39 cases) and just 45.45% (10 of 26 cases) for the ureteral tumours (**Table 1**).

**Table 1:** The most significant prognostic factors

		Recurrence
Location	Renal pelvis	21 (53.84%)
	Ureter	10 (45.45%)
Grade	Low grade	16 (36.36%)
	High grade	15 (71.42%)
Tumor size	< 1.5 cm	22 (43.13%)
	1.5 - 2 cm	9 (64.2%)
Overall recurrence		31 (47.69%)

**Fig. 7:**
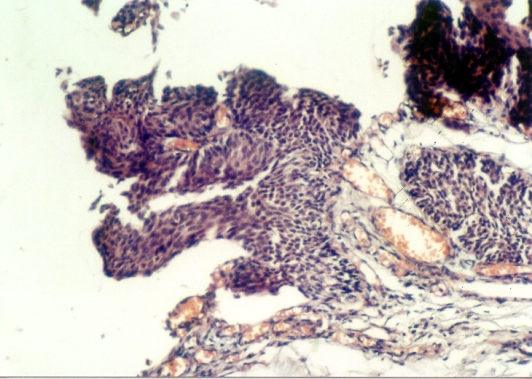
pT1G1 tumor – no recurrence after 36 months

The recurrence rate for low-grade tumours was of 36,36% (16 of 44 cases) and 71.42% (15 of 21 cases) for high-grade tumours (**Fig. 7**).

Regarding the tumour size, it is well known that there is a direct connection between size and stage. In our study the tumours over 1.5 cm were associated with a significant higher recurrence rate (especially local recurrence) compared with the tumours below 1.5 cm (64.2 versus 43.13%). 

Another prognosis factor was the personal history of bladder TCC. 6 out of the 9 patients with bladder TCC history had an upper urinary tract recurrence. 

## Discussion

The ureteroscopic and percutaneous approach of UUT-UCCs was first described in the mid 1980 [**2**]. Although there a small number of published studies regarding the role of the laser treatment in UUT-UCCs, their number is growing constantly. Most of them report a low progression rate (concerning grade and stage) for the low-grade tumours. The disease specific mortality rate for low-grade tumours is close to zero in most studies [**23**]. The first studies reported recurrence rates below 15% for the cases managed by endoscopic treatment, but with a small number of cases and short follow-up periods [**24**]. The recent studies describe local recurrence rates between 29 and 88%. These big differences between results are explained by the different and irregular designs of the studies. Most authors describe recurrence rates of 35-45% at upper urinary tract level and 30-36% for the bladder [**25,26**]. The recurrence rate from our study is 47.69% for the upper urinary tract, a value higher than the average rate published in literature, explainable by the relatively high number of cases from the study group with imperative indication for treatment.

In general the most important prognostic factors for the evolution of UUT-UCCs are the tumour location, grade and size. 

After some authors, the location is a less important prognostic factor, with small differences regarding the recurrence rates (33% for pyelocaliceal location versus 31% for ureteral tumours) [**27**]. Other authors believe that the pyelocaliceal location, with the bladder TCC history present, is the most important prognostic factors for the UUT-UCCs [**28**]. In our study group there are significant differences concerning the recurrence rate depending on location, this being a significant prognostic factor. 

The tumour size is an important prognostic factor and is strongly associated with the risk of recurrence. Generally, the endoscopic approach of tumours over 2 cm is not indicated, most of them being aggressive tumours, high-grade or invasive (T2-3). Also, the tumours between 1.5 and 2 cm have a worse prognosis than the tumours below 1.5 cm, no matter the approach: anterograde or retrograde [**28,29**]. As it was mentioned before, the results of this study are confirming the results published by other authors, proving the importance of the tumour size for the prognosis of UUT-UCCs treated endoscopically. 

The tumour grade is maybe the most important prognostic factor, the best results being obtained for the low-grade tumours. Thus, the disease specific survival rate at 5 years is variable in some studies from 80% for low grade tumours to 45% for high-grade, some authors questioning the need for nephroureterectomy in patients with bilateral functioning kidneys and low-grade tumours [**9,13,15,28**]. The current study proves that the main indication for the endoscopic approach of UUT-UCCs is represented by the low-grade tumours, with the lowest recurrence rate (36% versus 71%). On the contrary, for the high-grade lesions the endoscopic treatment has more a palliative intent than a curative one. 

These results underline the necessity for the overlook of the upper urinary tract and bladder after endoscopic treatment. In fact, the patients who choose the endoscopic treatment should be advised from the start to expect at least one recurrence. This is the reason why the follow-up protocol should include cystoscopy, cytology and ureteroscopy at 3 and 6 months in the first year after the endoscopic treatment of UUT-UCCs. 

## Conclusions

Endoscopic management of UUT-UCCs offers the advantage of preserving of renal function and may also be used in patients who would not tolerate invasive therapies. However, laser treatment of malignant urothelial lesions in the upper urinary tract should be reserved for a selected patient only. The initial stage and grade of the tumor is the key to defining the success rate. The patients treated by endoscopic surgery need careful surveillance due to frequent recurrences.

## References

[1] Munoz  JJ, Ellison  LM (2000). Upper tract urothelial neoplasms: incidence and survival during the last 2 decades. J Urol.

[2] Roupret  M, Zigeuner  R, Palou  J (2011). European Guidelines for the Diagnosis and Management of Upper Urinary Tract Urothelial Cell Carcinomas.

[3] Palou  J, Caparros  J, Orsola  A (1995). Transurethral resection of the intramural ureter as the first step of nephroureterectomy. J.Urol.

[4] Geavlete  P, Constantinescu  E, Georgescu  D (2009). Nephroureterectomy with endoscopic ureteral desinsertion in the treatment of upper urinary tract tumours – experience on 150 cases. J. Endourol.

[5] Geavlete  P, Multescu  R, Geavlete  B (2011). Bipolar plasma vaporization – an innovative intramural ureter detachment method during nephroureterectomy. Journal of Medicine and Life.

[6] Rassweiler  JJ, Schulze  M, Marrero  R (2004). Laparoscopic nephroureterectomy for upper urinary tract transitional cell carcinoma: is it better than open surgery?. Eur Urol.

[7] El Fettouh  HA, Rassweiler  JJ, Schulze  M (2002). Laparoscopic radical nephroureterectomy: results of an international multicenter study. Eur Urol.

[8] Lam  JS, Gupta  M (2004). Ureteroscopic management of upper tract transitional cell carcinoma. Urol Clin North Am.

[9] Lee  BR, Jabbour  ME, Marshall  FF (1999). 13-year survival comparison of percutaneous and open nephroureterectomy approaches for management of transitional cell carcinoma of renal collecting system: equivalent outcomes. J Endourol.

[10] Palou  J, Piovesan  LF, Huguet  J (2004). Percutaneous nephroscopic management of upper urinary tract transitional cell carcinoma: recurrence and long-term followup. J Urol.

[11] Grasso  M, Fraiman  M, Levine  M (1999). Ureteropyeloscopic diagnosis and treatment of upper urinary tract urothelial malignancies. Urology.

[12] Pasqui  F, Dubosq  F, Tchala  K (2004). Impact on active scope deflection and irrigation flow of all endoscopic working tools during flexible. Eur Urol.

[13] Elliott  DS, Segura  JW, Lightner  D (2001). Is nephroureterectomy necessary in all cases of upper tract transitional cell carcinoma? Long-term results of conservative endourologic management of upper tract transitional cell carcinoma in individuals with a normal contralateral kidney. Urology.

[14] Clark  PE, Streem  SB, Geisinger  MA (1999). 13-year experience with percutaneous management of upper tract transitional cell carcinoma. J Urol.

[15] Deligne  E, Colombel  M, Badet  L (2002). Conservative management of upper urinary tract tumours. Eur Urol.

[16] Martinez-Pineiro JA, Garcia Matres MJ, Martinez-Pineiro L (1996). Endourological treatment of upper tract urothelial carcinomas: analysis of a series of 59 tumours. J Urol.

[17] Greene  FL, Page  DL, Fleming  ID (2002). AJCC cancer staging manual. 6th ed.

[18] Epstein  JI, Amin  MB, Reuter  VR (1998). Bladder Consensus Conference Committee. The World Health Organization/International Society of Urological Pathology consensus classification of urothelial (transitional cell) neoplasms of the urinary bladder. Am J Surg Pathol.

[19] Liatsikos  EN, Dinlenc  CZ, Kapoor  R (2001). Transitional cell carcinoma of the renal pelvis: ureteroscopic and percutaneous approach. J Endourol.

[20] 20.Geavlete  P, Nita  Gh, Multescu  R (2008). Transurethral resection in ureteral tumours – long term follow-up. Eur Urol Suppl.

[21] Geavlete  P, Cauni  V, Mirciulescu  V (2008). Abordul percutanat în patologia aparatului urinar superior – experienţa Clinicii de Urologie a Spitalului Clinic de Urgenţă „Sf. Ioan” Bucureşti.. Revista Română de Urologie.

[22] Huffman  JL, Bagley  DS, Lyon  ES (1985). Endoscopic diagnosis and treatment of upper-tract urothelial tumours. A preliminary report.. Cancer.

[23] Johnson  GB, Grasso  M (2005). Ureteroscopic management of upper urinary tract transitional cell carcinoma. Curr Opin Urol.

[24] Gerber  G, Lyon  E (1993). Endourological management of upper tract urothelial tumours. J. Urol.

[25] Razdan  S, Johannes  J, Cox  M (2005). Current practice patterns in urologic management of upper-tract transitional cell carcinoma. J Endourol.

[26] Bader  M, Sroka  R, Gratzke  C (2009). Laser therapy for upper urinary tract transitionall cell carcinoma: indications and management. Eur Urol.

[27] Tawfiek  ER, Bagley  DH (1999). Upper-tract transitional cell carcinoma. Urology.

[28] Iborra  I, Solsona  J, Casanova  J (2003). Conservative elective treatment of upper urinary tract tumours: a multivariate analysis of prognostic factors for recurrence and progression. J Urol.

[29] Johnson  GB, Fraiman  M, Grasso  M (2005). Broadening experience with the retrograde endoscopic management of upper urinary tract urothelial malignancies. BJU Int.

